# Neddylation as a molecular integrator of metabolic dysfunction and endothelial dysfunction in type 2 diabetes

**DOI:** 10.1038/s41401-026-01910-w

**Published:** 2026-08-24

**Authors:** Zhongjian Cheng, Chris Wittmann, Raj Kishore

**Affiliations:** 1 Aging + Cardiovascular Discovery Center, Department of Cardiovascular Sciences, Lewis Katz School of Medicine Temple University, Philadelphia, PA, USA

**Keywords:** neddylation, NEDD8, endothelial dysfunction, type 2 diabetes mellitus, metabolic dysfunction

## Abstract

Type 2 diabetes mellitus (T2D) is a major global health challenge associated with a high burden of cardiovascular and microvascular complications. Endothelial dysfunction is a hallmark of T2D and represents a critical link between metabolic abnormalities and vascular disease. Chronic hyperglycemia, insulin resistance, obesity-associated inflammation, oxidative stress, and mitochondrial dysfunction disrupt endothelial homeostasis, resulting in impaired nitric oxide bioavailability, vascular inflammation, endothelial senescence, barrier dysfunction, and defective vascular repair. However, the molecular mechanisms integrating metabolic stress with endothelial injury in T2D remain incompletely understood. Neddylation is a reversible ubiquitin-like post-translational modification mediated by neural precursor cell expressed, developmentally downregulated 8 (NEDD8). Through activation of Cullin-RING E3 ubiquitin ligases and modification of non-cullin substrates, neddylation regulates protein turnover, signal transduction, metabolism, inflammation, oxidative stress responses, and mitochondrial homeostasis. Emerging evidence suggests that dysregulated neddylation contributes to several metabolic abnormalities implicated in endothelial dysfunction, including insulin resistance, obesity-associated metabolic dysfunction, oxidative stress, and mitochondrial dysfunction. In diabetic retinopathy, excessive cullin neddylation promotes degradation of protective factors such as nuclear factor erythroid 2-related factor 2 (NRF2) and heterogeneous nuclear ribonucleoprotein A2/B1 (hnRNPA2B1), thereby enhancing oxidative stress, inflammation, vascular leakage, and pathological angiogenesis. Conversely, physiological neddylation appears necessary for endothelial homeostasis through regulation of endothelial barrier integrity, angiogenesis, and stabilization of vascular endothelial growth factor receptor 2 (VEGFR2). Although direct evidence remains largely limited to retinal endothelial cells and diabetic retinopathy models, current findings support the hypothesis that dysregulated neddylation may act as an important molecular link between metabolic dysfunction and endothelial injury in diabetes. Pharmacological modulation of the neddylation pathway, including inhibition of the NEDD8-activating enzyme with MLN4924 (pevonedistat), improves metabolic homeostasis, suppresses inflammatory signaling, enhances antioxidant defenses, and attenuates vascular injury in preclinical studies. In this review, we summarize current advances in neddylation biology, discuss emerging evidence linking dysregulated neddylation to diabetes-associated endothelial dysfunction, highlight critical knowledge gaps, and evaluate the opportunities and challenges of targeting the neddylation pathway for the prevention and treatment of diabetic vascular complications.

## INTRODUCTION

Type 2 diabetes mellitus (T2D) has become one of the most pressing public health challenges worldwide, with prevalence continuing to rise in parallel with increasing rates of obesity, population aging, and sedentary lifestyles. According to the International Diabetes Federation, approximately 537 million adults were living with diabetes in 2021, and this number is projected to reach 783 million by 2045, with T2D accounting for nearly 90%–95% of all cases [1]. The growing global burden of diabetes is accompanied by substantial healthcare expenditures and a marked increase in cardiovascular morbidity and mortality, making the identification of novel mechanisms underlying disease progression a major research priority [1].

Although hyperglycemia is the defining metabolic feature of diabetes, vascular complications represent the principal cause of disability and death among affected individuals. Endothelial dysfunction is widely recognized as an early and critical event in the pathogenesis of both microvascular complications, including diabetic retinopathy, nephropathy, and neuropathy, and macrovascular disorders such as atherosclerosis, coronary artery disease, and peripheral artery disease [2–6]. The vascular endothelium is a highly specialized organ that regulates vascular tone, permeability, thrombosis, inflammation, and angiogenesis through the coordinated production of vasoactive mediators, including nitric oxide (NO), prostacyclin, and endothelin-1 [7–12]. Under diabetic conditions, chronic exposure to hyperglycemia, insulin resistance, dyslipidemia, and inflammatory stimuli disrupts endothelial homeostasis, leading to reduced NO bioavailability, oxidative stress, vascular inflammation, and impaired reparative capacity [3, 5, 6, 13, 14].

The molecular pathways linking metabolic dysfunction to endothelial injury are complex and involve extensive regulation at both transcriptional and post-translational levels. In recent years, post-translational modifications have emerged as critical mediators of cellular adaptation to metabolic stress by influencing protein stability, localization, and signaling activity [15]. Among these modifications, neddylation has attracted considerable attention because of its central role in regulating protein turnover through activation of Cullin-RING E3 ubiquitin ligases (CRLs), the largest family of E3 ubiquitin ligases in mammalian cells [16–18]. Neddylation is a reversible ubiquitin-like modification involving covalent conjugation of neural precursor cell expressed, developmentally downregulated 8 (NEDD8) to target proteins through a highly conserved enzymatic cascade consisting of E1 activating, E2 conjugating, and E3 ligating enzymes [19–27]. Through regulation of CRLs and an expanding repertoire of non-cullin substrates, neddylation influences numerous biological processes, including cell-cycle progression, inflammation, oxidative stress responses, metabolism, mitochondrial function, and cellular senescence [15, 17, 27].

Accumulating evidence suggests that dysregulated neddylation contributes to the pathogenesis of a broad range of human diseases. While initially studied primarily in cancer biology, recent investigations have implicated aberrant neddylation signaling in neurodegenerative disorders, inflammatory diseases, cardiovascular pathology, and metabolic dysfunction [15, 28]. Emerging studies demonstrate that components of the neddylation machinery are altered in obesity, nonalcoholic fatty liver disease, and T2D, where they regulate key pathways involved in insulin signaling, glucose metabolism, lipid homeostasis, inflammatory activation, and oxidative stress [29–34]. Importantly, pharmacological inhibition of the NEDD8 pathway using NEDD8-activating enzyme (NAE) inhibitors such as MLN4924 (pevonedistat) has shown beneficial effects in multiple experimental models by improving metabolic homeostasis, attenuating inflammation, and reducing tissue injury [35–39].

Recent studies further suggest that neddylation plays a previously underappreciated role in vascular biology. Components of the neddylation pathway are expressed in endothelial cells (ECs) and participate in the regulation of angiogenesis, endothelial barrier integrity, vascular remodeling, inflammatory signaling, and senescence [40–46]. Under pathological conditions, excessive neddylation promotes endothelial dysfunction through degradation of protective proteins, activation of inflammatory pathways, and disruption of redox homeostasis [18, 31, 47, 48]. In particular, studies in diabetic retinopathy have demonstrated that increased cullin neddylation exacerbates oxidative stress and vascular injury through suppression of Nuclear factor erythroid 2-related factor 2 (NRF2)-dependent antioxidant signaling and degradation of endothelial protective factors [18, 47, 48]. These findings raise the possibility that dysregulated neddylation may serve as a mechanistic link connecting metabolic stress to endothelial injury across multiple diabetic vascular complications.

Notably, many of the major drivers of endothelial dysfunction in T2D—including hyperglycemia, insulin resistance, obesity-associated inflammation, oxidative stress, and mitochondrial dysfunction—are regulated by neddylation-dependent signaling pathways [29–35, 49–52]. We therefore propose that dysregulated neddylation functions as a molecular integrator linking metabolic stress to endothelial dysfunction in T2D. However, despite growing interest in the field, our understanding of the role of neddylation in diabetes-associated endothelial dysfunction remains incomplete, and many mechanistic questions remain unresolved.

In this review, we summarize current advances in the biology of the neddylation pathway and discuss emerging evidence implicating neddylation in diabetes and endothelial dysfunction. We highlight the role of neddylation in major metabolic drivers of vascular injury, including hyperglycemia, insulin resistance, obesity, oxidative stress, and mitochondrial dysfunction, and evaluate its therapeutic potential as a novel target for preventing and treating diabetes-associated vascular complications.

## BIOLOGY OF THE NEDDYLATION PATHWAY

Neddylation is an evolutionarily conserved post-translational modification in which NEDD8, a ubiquitin-like protein, is covalently attached to specific lysine residues on target proteins [19, 20]. Similar to ubiquitination, neddylation regulates protein stability, activity, subcellular localization, and protein-protein interactions, thereby influencing diverse cellular processes [19, 20]. Unlike ubiquitination, however, its primary function is not to directly target proteins for degradation but rather to modulate signaling networks and ubiquitin ligase activity [20, 21]. Neddylation is mediated through an enzymatic cascade involving the NEDD8-activating enzyme (NAE), the E2 conjugating enzymes UBE2M and UBE2F, and several E3 ligases, including RBX1 and RBX2 (Figs. 1 and 2) [22–26]. Because NAE initiates the neddylation cascade, it represents the principal upstream regulatory node and the major pharmacological target of current neddylation inhibitors.

The best-characterized substrates of neddylation are members of the cullin protein family (CUL1–5, CUL7, and CUL9), which function as scaffold proteins for Cullin-RING ubiquitin ligases (CRLs), the largest family of E3 ubiquitin ligases in mammalian cells [21, 26]. Neddylation activates CRLs and thereby regulates the ubiquitination and turnover of hundreds of proteins involved in cell-cycle progression, DNA repair, inflammation, oxidative stress responses, metabolism, apoptosis, and cellular adaptation to stress [15–17]. Consequently, cullin neddylation represents a central signaling hub linking protein turnover to cellular homeostasis. Among cullin family members, CUL3 has emerged as a particularly important regulator of metabolic and redox homeostasis through its control of the KEAP1–NRF2 antioxidant pathway, insulin sensitivity, and mitochondrial function [29, 35].

Beyond cullins, an expanding repertoire of non-cullin substrates has considerably broadened the biological scope of NEDD8 signaling [15, 17, 27]. Importantly, neddylation can regulate cellular function through both direct conjugation of NEDD8 to non-cullin substrates and indirect mechanisms mediated by activation of Cullin-RING ligases (CRLs), which subsequently control the ubiquitination and degradation of downstream target proteins.

Neddylation has been identified on transcription factors, metabolic enzymes, receptor-associated proteins, and stressresponse regulators, allowing direct regulation of protein activity, signaling, and intracellular trafficking independently of CRL-mediated protein degradation [15, 27]. These findings have transformed the view of neddylation from a pathway dedicated primarily to cullin regulation into a versatile signaling network analogous to phosphorylation or SUMOylation [15, 27].

Recent studies have identified several neddylation-regulated pathways that are particularly relevant to metabolic disease. Neddylation directly regulates phosphoenolpyruvate carboxykinase 1 (PCK1), a rate-limiting enzyme in gluconeogenesis, thereby linking NEDD8 signaling to glucose metabolism [30]. Neddylation also influences insulin signaling through regulation of insulin receptor substrate stability and controls oxidative stress through modulation of the CUL3–KEAP1–NRF2 pathway [29, 35]. In addition, UBE2M-dependent neddylation of TRIM21 promotes obesity-associated inflammation and metabolic dysfunction, highlighting a role for neddylation in immunometabolic regulation [31]. Beyond these cullin-dependent pathways, emerging evidence demonstrates that neddylation also regulates non-cullin substrates, including VEGFR2 and hnRNPA2B1, thereby influencing endothelial homeostasis, angiogenesis, senescence, and vascular barrier function [46–48]. Together, these observations position neddylation at the intersection of glucose metabolism, insulin signaling, inflammation, oxidative stress, mitochondrial homeostasis, and endothelial biology, pathways that are central to the development of endothelial dysfunction and diabetic vascular disease.

Like other post-translational modifications, neddylation is highly dynamic and reversible. Deneddylation is mediated primarily by the COP9 signalosome (CSN), which removes NEDD8 from cullins and regulates CRL recycling, and by NEDD8-specific proteases such as NEDP1/SENP8 that remove NEDD8 from both cullin and non-cullin substrates [20, 53–56]. Maintenance of the balance between neddylation and deneddylation is therefore critical for normal cellular function, whereas disruption of this equilibrium contributes to cancer, cardiovascular disease, neurodegenerative disorders, and metabolic dysfunction.

The central role of NAE in initiating the neddylation cascade has made it an attractive therapeutic target. Pharmacological inhibition of NAE with MLN4924 (pevonedistat) or the second-generation inhibitor TAS4464 suppresses global neddylation signaling and has demonstrated beneficial effects in experimental models of cancer and metabolic disease [38, 39, 57]. In addition to NAE inhibition, increasing efforts are focused on selectively targeting UBE2M-, UBE2F-, and DCN1-dependent pathways to achieve greater specificity while minimizing systemic toxicity [25, 58]. Because neddylation regulates multiple processes implicated in diabetes, including insulin signaling, inflammation, oxidative stress, mitochondrial metabolism, and endothelial homeostasis, modulation of this pathway has emerged as a promising therapeutic strategy for diabetes-associated vascular complications [29–35, 57, 59]. However, physiological neddylation is also required for endothelial integrity, angiogenesis, mitochondrial function, and tissue repair, indicating that future therapies will likely require selective, tissue-specific, or substrate-specific approaches that suppress pathological neddylation while preserving essential physiological functions [40, 41, 44–46].

Recent human genetic studies further underscore the physiological importance of intact neddylation signaling. Individuals carrying biallelic pathogenic variants in NAE1, which encodes the regulatory subunit of the NEDD8-activating enzyme, develop a multisystem disorder characterized by intellectual disability, neurodevelopmental abnormalities, neurodegeneration, immune dysfunction, stress-mediated lymphopenia, and skeletal defects [60]. Functional analyses demonstrated impaired cellular neddylation and increased susceptibility to cellular stress, providing direct evidence that disruption of the NEDD8 pathway causes human disease [60]. In addition, genomic and transcriptomic studies have identified genetic and expression abnormalities involving multiple components of the neddylation machinery, including *NEDD8, NAE1, UBA3, UBE2M, UBE2F, RBX1*, and *RNF7*, across diverse human diseases [15, 60, 61]. Although direct genetic associations between neddylation pathway variants and diabetes-associated endothelial dysfunction have not yet been established, these findings provide important human evidence supporting the critical physiological role of intact neddylation signaling and highlight the need for future studies investigating the contribution of neddylation-related genetic variation to cardiometabolic and vascular diseases (Figs. 1 and 2) [60, 61].

## ENDOTHELIAL DYSFUNCTION IN DIABETES

Endothelial dysfunction is a central feature of T2D and represents a key mechanistic link between metabolic abnormalities and diabetic vascular complications [13, 14]. Chronic hyperglycemia, insulin resistance, obesity-associated inflammation, oxidative stress, and mitochondrial dysfunction impair endothelial homeostasis, resulting in reduced nitric oxide bioavailability, endothelial activation, increased vascular permeability, senescence, and defective vascular repair [13, 14, 62–69]. These alterations contribute to the development of both microvascular complications, including retinopathy and nephropathy, and macrovascular diseases such as atherosclerosis and peripheral artery disease [2–6, 13, 14, 62, 63]. Emerging evidence further implicates extracellular vesicle-mediated signaling, epigenetic regulation, and impaired progenitor cell function in the progression of diabetic vascular injury [13, 62, 70–72]. Because many of these pathways are regulated by neddylation, understanding the role of NEDD8 signaling may provide important insights into the mechanisms linking metabolic dysfunction to endothelial injury in diabetes [15, 29–35, 49–52].

## NEDDYLATION IN DIABETES-ASSOCIATED ENDOTHELIAL DYSFUNCTION

### Physiological neddylation in endothelial homeostasis

Neddylation is a highly conserved post-translational modification that plays a fundamental role in maintaining vascular homeostasis through the regulation of protein stability, signal transduction, cellular stress responses, and endothelial function [15, 40, 41]. Components of the neddylation machinery, including NAE1, UBA3, UBE2M, RBX1, SAG/RBX2, and DCN1, are expressed and functionally active in ECs, highlighting the importance of this pathway in vascular biology [42, 43]. Physiological neddylation is required for endothelial survival, angiogenesis, vascular remodeling, and maintenance of endothelial barrier integrity. Consistent with this role, pharmacological inhibition of neddylation using the NEDD8-activating enzyme inhibitor MLN4924 suppresses endothelial tube formation and angiogenesis both in vitro and in vivo, demonstrating that tightly regulated neddylation is indispensable for normal vascular development and endothelial function [41, 44, 45]. Recent evidence has further revealed an important role for neddylation in preventing endothelial senescence. Recent work identified VEGFR2 as a direct non-cullin substrate of neddylation and demonstrated that UBE2M-mediated VEGFR2 neddylation preserves endothelial homeostasis by maintaining endothelial proliferative and angiogenic capacity and protecting pulmonary vascular ECs from premature senescence [46]. Disruption of UBE2M-dependent neddylation reduced VEGFR2 stability and accelerated endothelial senescence, whereas preservation of VEGFR2 neddylation maintained endothelial proliferative and angiogenic capacity [46]. Because VEGFR2 is a master regulator of endothelial survival, migration, angiogenesis, and vascular repair, these findings suggest that physiological neddylation directly supports endothelial regenerative function independently of its classical role in regulating CRL [46]. Moreover, the identification of VEGFR2 as a non-canonical neddylation substrate broadens our understanding of endothelial neddylation signaling and highlights the importance of substrate-specific regulation in vascular biology. These observations further emphasize that complete inhibition of neddylation may have unintended consequences on endothelial health by disrupting VEGFR2-dependent adaptive and reparative responses.

Mechanistically, neddylation regulates the turnover of proteins involved in cytoskeletal organization and endothelial barrier maintenance. One such target is the small GTPase RhoA, a substrate of CRL3 that controls actin cytoskeletal dynamics and endothelial morphogenesis [44, 73]. Inhibition of neddylation results in RhoA accumulation, cytoskeletal disorganization, and impaired endothelial morphogenesis, whereas RhoA knockdown rescues these defects [44, 73]. In addition, neddylated CUL3 is required for proper localization of vascular endothelial cadherin (VE-cadherin) at intercellular junctions and for preservation of endothelial barrier function [40]. Pharmacological inhibition of neddylation by MLN4924 or knockdown of CUL3 disrupts VE-cadherin (also known as cadherin-5 and CD144)-mediated cell-cell adhesion and significantly increases endothelial permeability [40]. Likewise, endothelial-specific deletion of SAG/RBX2 impairs endothelial proliferation, migration, tube formation, angiogenesis, and vascular remodeling [41].

Collectively, these findings demonstrate that physiological neddylation is essential for maintaining endothelial integrity, barrier function, angiogenesis, vascular remodeling, and protection against endothelial senescence. Through regulation of both canonical cullin-dependent pathways and non-canonical substrates such as VEGFR2, neddylation preserves endothelial homeostasis and vascular health, whereas disruption of this pathway may predispose ECs to dysfunction, impaired repair, and vascular disease.

### Overactive neddylation in diabetes-associated endothelial dysfunction

Although physiological neddylation is essential for endothelial homeostasis, excessive or dysregulated neddylation contributes to endothelial dysfunction under pathological conditions. One of the earliest studies linking neddylation to vascular injury demonstrated that oxidized low-density lipoprotein (OxLDL), a major atherogenic stimulus, markedly increases protein neddylation in ECs and vascular tissues [74]. Enhanced neddylation promotes degradation of histone deacetylase 2 (HDAC2), leading to increased arginase-2 expression, reduced eNOS-derived NO production, and impaired endothelium-dependent vasorelaxation. required for endothelial integrity [74]. Importantly, inhibition of neddylation with MLN4924 restores endothelial function and vascular relaxation, implicating excessive neddylation as a direct mediator of vascular injury [74]. Furthermore, inflammatory stimuli such as lipopolysaccharide (LPS) induce Cullin-dependent activation of NF-κB signaling in ECs, whereas inhibition of neddylation suppresses inflammatory signaling and cytokine production [42, 56]. Consistently, overexpression of the deneddylase COP9 signalosome subunit 5 (CSN5) attenuates NF-κB activation and monocyte adhesion, supporting a pathogenic role for excessive neddylation in vascular inflammation [75].

Accumulating evidence indicates that hyperactive neddylation is particularly relevant in diabetes-associated endothelial dysfunction. Hyperglycemia and AGE-associated signaling have been reported to enhance neddylation pathway activity and CRL function, resulting in accelerated degradation of proteins that normally protect ECs from oxidative stress and inflammation [18, 48]. In diabetic retinopathy, increased CUL3 neddylation promotes ubiquitination and degradation of NRF2, suppressing antioxidant defenses and increasing ROS accumulation, thereby contributing to oxidative stress, inflammation, blood-retinal barrier disruption, and microvascular injury [18, 48]. Pharmacological inhibition of neddylation stabilizes NRF2, restores antioxidant responses, and preserves endothelial barrier integrity, highlighting the pathogenic role of excessive neddylation in diabetic vascular disease [18, 48].

Additional studies have identified heterogeneous nuclear ribonucleoprotein A2B1 (hnRNPA2B1) as another protective target of dysregulated neddylation. High-glucose exposure promotes neddylation-mediated degradation of hnRNPA2B1, disrupting the miR-93-5p/VEGFA signaling axis and resulting in endothelial dysfunction, vascular leakage, and pathological retinal angiogenesis [47]. Restoration of either hnRNPA2B1 or miR-93-5p significantly attenuates retinal vascular injury in diabetic models [47]. It remains unknown whether high glucose promotes hnRNPA2B1 degradation through cullin neddylation-dependent activation of CRLs or through alternative non-canonical neddylation mechanisms. Similarly, mesenchymal stem cell-derived extracellular vesicles enriched in miR-143-3p protect against diabetic retinopathy by suppressing CUL3 neddylation, restoring NRF2 signaling, and reducing oxidative stress-mediated endothelial injury [48]. Together, these findings identify NRF2 and hnRNPA2B1 as important neddylation-sensitive protective factors whose degradation contributes to diabetes-induced endothelial dysfunction [18, 47, 48].

Notably, the current understanding of neddylation in diabetes-impaired endothelial function is derived predominantly from studies of retinal ECs and diabetic retinopathy models [18, 47, 48]. These studies have provided important mechanistic insights into how excessive neddylation promotes oxidative stress, inflammation, vascular leakage, and pathological angiogenesis through degradation of protective factors such as NRF2 and hnRNPA2B1 [18, 47, 48]. However, ECs exhibit substantial heterogeneity across vascular beds, and the molecular mechanisms governing retinal endothelial dysfunction may not fully reflect those operating in other vascular territories, including the coronary, aortic, renal, and peripheral microvascular endothelium [13, 68]. Given that endothelial dysfunction is a central contributor to diabetic cardiovascular disease, atherosclerosis, peripheral artery disease, nephropathy, and impaired wound healing, further studies are needed to determine whether dysregulated neddylation similarly contributes to endothelial injury in these vascular beds [13, 68]. Elucidating the EC-specific roles of neddylation across different diabetic complications will not only improve our understanding of disease pathogenesis but may also facilitate the development of targeted therapeutic strategies aimed at preventing diabetes-associated vascular and cardiovascular complications (Table 1).

The identification of VEGFR2 as a protective neddylation substrate further underscores the context-dependent nature of NEDD8 signaling in the endothelium [46]. Whereas excessive neddylation promotes degradation of protective factors such as NRF2 and hnRNPA2B1 under diabetic conditions, physiological neddylation appears necessary to maintain VEGFR2 stability and suppress endothelial senescence [46]. These findings suggest that the vascular consequences of neddylation are highly substrate specific and occur through distinct mechanisms. Consequently, future therapeutic strategies may need to selectively target pathological neddylation events while preserving physiological neddylation pathways required for endothelial maintenance and vascular repair.

Collectively, current evidence supports a model in which physiological neddylation maintains endothelial homeostasis, whereas excessive neddylation induced by diabetic stimuli promotes degradation of protective proteins such as NRF2 and hnRNPA2B1, leading to oxidative stress, inflammation, endothelial barrier dysfunction, vascular leakage, and pathological angiogenesis [18, 47, 48]. These findings identify neddylation as a potential molecular link between metabolic stress and vascular injury in diabetes and suggest that modulation of the neddylation pathway may represent a promising therapeutic strategy for preventing diabetes-associated vascular complications [13, 68].

## NEDDYLATION IN METABOLIC DRIVERS OF ENDOTHELIAL DYSFUNCTION IN DIABETES

Importantly, much of the current evidence linking neddylation to glucose metabolism, insulin resistance, obesity-associated metabolic dysfunction, and mitochondrial homeostasis has been derived from studies in hepatocytes, adipocytes, macrophages, skeletal muscle cells, and cancer cells rather than endothelial cells, and therefore, the relevance of these mechanisms to endothelial dysfunction requires further validation.

Endothelial dysfunction is a hallmark of diabetes and a key driver of both microvascular and macrovascular complications. In addition to direct endothelial injury induced by hyperglycemia, multiple metabolic abnormalities—including insulin resistance, obesity-associated inflammation, lipotoxicity, oxidative stress, and mitochondrial dysfunction—have been identified as major metabolic drivers of endothelial dysfunction in diabetes [5, 13, 66–68]. These interconnected disturbances promote endothelial activation, impaired NO bioavailability, inflammation, oxidative stress, and vascular remodeling, ultimately accelerating the development and progression of diabetic vascular disease [5, 13, 66–68]. Recent evidence suggests that neddylation regulates many of the pathways underlying these metabolic drivers of vascular injury. Beyond its direct effects on ECs, neddylation modulates glucose homeostasis, insulin signaling, lipid metabolism, inflammatory responses, redox balance, and mitochondrial function [29, 30, 33, 35, 51, 52, 76, 77]. Consistent with the concept proposed above, dysregulated neddylation may contribute to endothelial dysfunction through coordinated effects on glucose metabolism, insulin signaling, inflammation, oxidative stress, and mitochondrial homeostasis [13, 68]. Furthermore, experimental studies have demonstrated that inhibition of neddylation improves metabolic homeostasis, reduces oxidative stress and inflammation, enhances angiogenesis and tissue repair, and ameliorates vascular injury in several diabetic disease models [33, 35, 37, 52]. Although the direct involvement of neddylation in diabetes-associated endothelial dysfunction remains incompletely understood, increasing evidence indicates that dysregulated neddylation contributes to several major metabolic abnormalities that drive endothelial injury. Therefore, understanding how neddylation regulates hyperglycemia, insulin resistance, obesity-associated metabolic dysfunction, oxidative stress, and mitochondrial homeostasis may provide important mechanistic insights into how these metabolic drivers contribute to diabetic vascular disease and may uncover novel therapeutic opportunities for preventing diabetes-associated endothelial dysfunction and cardiovascular complications.

### Neddylation in glucose metabolism and insulin resistance

Emerging evidence identifies neddylation as an important regulator of glucose homeostasis and insulin signaling. Neddylation influences the stability and activity of proteins involved in insulin receptor signaling, glucose metabolism, and pancreatic β-cell function, thereby contributing to both hyperglycemia and insulin resistance[29, 34, 35, 51].

A major mechanism involves the regulation of insulin receptor substrate (IRS) proteins. Pharmacological inhibition of cullin neddylation using MLN4924 enhances hepatic insulin signaling by preventing CRL-mediated degradation of IRS proteins, thereby prolonging insulin action and improving insulin sensitivity [35]. Consistent with this finding, knockdown of CUL3, but not CUL1, stabilizes IRS proteins and improves insulin signaling in the livers of high-fat diet-fed mice, highlighting a specific role for CUL3-dependent neddylation in regulating insulin responsiveness [35]. Furthermore, neddylation of IRS proteins has been identified as a bona fide regulatory mechanism controlling insulin receptor signaling, providing direct evidence that the NEDD8 pathway modulates insulin action [51]. In addition, selective inhibition of CUL3-dependent neddylation enhances glucose-stimulated insulin secretion from pancreatic β-cells and improves glycemic control in experimental models [29].

Neddylation also regulates glucose metabolism through additional mechanisms. Neddylation of phosphoenolpyruvate carboxykinase 1 (PCK1), a rate-limiting enzyme in gluconeogenesis, directly influences glucose homeostasis and metabolic adaptation [30]. Consistent with these observations, inhibition of neddylation suppresses hepatic glucose production, enhances peripheral glucose utilization, and attenuates hyperglycemia [29, 34, 35].

However, the relationship between neddylation and insulin sensitivity appears to be context-dependent. While partial inhibition of CUL3 activity improves insulin signaling, complete genetic inactivation of CUL3-RING E3 ligase activity induces NRF2-dependent reductive stress, mitochondrial dysfunction, hepatic lipodystrophy, and systemic insulin resistance [29]. These findings indicate that the metabolic consequences of neddylation modulation are highly context-dependent. While excessive neddylation may promote metabolic dysfunction under conditions of nutrient excess, basal CUL3-dependent neddylation is required for maintaining normal metabolic and redox homeostasis [29].

Collectively, current evidence indicates that neddylation is a critical regulator of glucose metabolism and insulin signaling through its effects on IRS stability, β-cell function, gluconeogenesis, and metabolic homeostasis [29, 30, 34, 35, 51, 78]. Because hyperglycemia and insulin resistance are major drivers of endothelial dysfunction in diabetes, dysregulated neddylation may represent an important mechanistic link between metabolic stress and vascular injury. Moreover, modulation of the NEDD8 pathway has shown promising glucose-lowering and insulinsensitizing effects in preclinical studies, highlighting its potential as a therapeutic target for T2D and its vascular complications [15, 34, 78].

### Neddylation in obesity-associated metabolic dysfunction

Emerging evidence identifies neddylation as an important regulator of obesity-associated metabolic dysfunction. Increased expression of NEDD8 and multiple components of the neddylation machinery, including NAE1, UBE2M, and neddylated cullins, has been observed in obesity, nonalcoholic fatty liver disease (NAFLD), and T2D, suggesting that activation of the neddylation pathway is a common feature of metabolic disease [30–33, 35, 52].

One of the best-characterized mechanisms linking neddylation to obesity involves regulation of peroxisome proliferator-activated receptor-γ (PPARγ), a master regulator of adipocyte differentiation and lipid storage. Neddylation stabilizes PPARγ and promotes adipogenesis, whereas pharmacological inhibition of neddylation suppresses adipocyte differentiation, reduces lipid accumulation, and attenuates HFD-induced obesity in mice [32]. Neddylation has also been implicated in inhibiting adipose tissue browning and thermogenesis, thereby favoring energy storage over energy expenditure [32].

The liver represents another major target of neddylation-dependent metabolic regulation. Hepatic neddylation and circulating NEDD8 levels are increased in both experimental and human NAFLD and correlate with disease severity [33]. Pharmacological inhibition of neddylation alleviates hepatic steatosis by enhancing mitochondrial fatty acid oxidation and promoting lipid catabolism through the DEPTOR–mTOR pathway [33, 52]. These findings suggest that excessive neddylation contributes to hepatic lipid accumulation and metabolic dysfunction.

Neddylation also regulates obesity-associated inflammation. UBE2M-mediated neddylation of TRIM21 promotes VHL degradation, HIF-1α stabilization, and IL-1β production, thereby amplifying inflammatory responses in adipose tissue and liver [31]. Consistent with this mechanism, macrophage-specific deletion of UBE2M attenuates obesity, hepatic steatosis, insulin resistance, and adipose tissue inflammation in HFD-fed mice [31].

Additional studies indicate that neddylation regulates key metabolic pathways involved in glucose and lipid homeostasis. Neddylation of phosphoenolpyruvate carboxykinase 1 (PCK1) modulates glucose metabolism, whereas loss of CUL3 activity induces NRF2-dependent reductive stress, hepatic lipodystrophy, and systemic insulin resistance [29, 30]. These findings suggest that both excessive and insufficient neddylation can disrupt metabolic homeostasis, highlighting the importance of maintaining balanced pathway activity [15, 29].

Collectively, current evidence supports a role for neddylation in obesity-associated metabolic dysfunction through regulation of adipogenesis, lipid metabolism, inflammation, and redox homeostasis [15, 29–35, 52]. Because these processes are major drivers of endothelial dysfunction in diabetes, dysregulated neddylation may represent an important mechanistic link between obesity-associated metabolic stress and vascular injury. Moreover, pharmacological inhibition of neddylation has demonstrated beneficial metabolic effects in preclinical studies, suggesting that selective modulation of the NEDD8 pathway may represent a promising therapeutic strategy for obesity, NAFLD, insulin resistance, and T2D [15, 31–34, 52].

### Neddylation in oxidative stress and mitochondrial homeostasis

Oxidative stress and mitochondrial dysfunction are closely interconnected mechanisms that contribute to endothelial dysfunction and diabetic vascular complications. Although ECs rely predominantly on glycolysis for ATP production, mitochondria remain critical regulators of reactive oxygen species (ROS) homeostasis, intracellular signaling, angiogenesis, and cell survival [69, 79–83]. Under diabetic conditions, excessive mitochondrial ROS production, impaired oxidative phosphorylation, dysregulated mitochondrial dynamics, and defective mitophagy contribute to reduced NO bioavailability, endothelial senescence, and vascular injury [69, 80–85].

Emerging evidence identifies neddylation as an important regulator of both cellular redox balance and mitochondrial homeostasis. One of the best-characterized pathways is the KEAP1–CUL3–RBX1–NRF2 axis, in which cullin neddylation controls the activity of the E3 ubiquitin ligase complex responsible for NRF2 degradation [15, 86–88]. Because NRF2 is a master regulator of antioxidant defense, alterations in neddylation can profoundly influence oxidative stress responses. Supporting this concept, hepatocyte-specific deletion of CUL3 induces persistent NRF2 activation, NADH reductive stress, mitochondrial dysfunction, hepatic lipodystrophy, and systemic insulin resistance, demonstrating that balanced neddylation is essential for redox and metabolic homeostasis [29].

Neddylation also directly regulates mitochondrial function and quality control. Genetic depletion of UBA3 or pharmacological inhibition of neddylation alters mitochondrial morphology, respiration, and metabolic programming, whereas hepatocyte-specific deletion of UBA3 impairs mitochondrial oxidative phosphorylation and fatty acid β-oxidation through destabilization of electron transfer flavoproteins [36, 50]. In addition, components of CRL participate in mitochondrial quality-control pathways. RBX1 regulates mitochondrial ROS production and mitophagy, whereas RBX2/SAG promotes mitochondrial homeostasis through stabilization of the mitophagy regulator PINK1 [89–91]. Collectively, these findings indicate that intact neddylation signaling is required for maintaining mitochondrial bioenergetics, mitophagy, and adaptation to metabolic stress [36, 89–95].

Additional evidence suggests that neddylation regulates oxidative stress through inflammatory signaling pathways. Pharmacological inhibition of neddylation suppresses proinflammatory cytokine production, whereas UBE2M-mediated neddylation of TRIM21 promotes obesity-associated inflammation and metabolic dysfunction [31, 77]. Because inflammation and ROS generation are tightly interconnected, dysregulated neddylation may contribute to a feed-forward cycle of oxidative stress and inflammatory activation during metabolic disease [15, 31, 34, 77].

Pharmacological inhibition of neddylation with MLN4924 enhances NRF2 accumulation, attenuates oxidative injury, and improves mitochondrial fatty acid oxidation in several experimental models [33, 52, 96–98]. These findings support the concept that neddylation regulates cellular adaptation to oxidative and metabolic stress through coordinated effects on antioxidant responses, mitochondrial metabolism, and inflammatory signaling.

Given that oxidative stress, impaired mitochondrial quality control, and excessive ROS generation are central drivers of diabetic endothelial dysfunction, neddylation may function as an upstream regulator linking metabolic stress to endothelial injury [69, 80–85]. Although direct evidence in diabetic ECs remains limited, current findings support the hypothesis that dysregulated neddylation contributes to endothelial dysfunction through disruption of NRF2 signaling, mitochondrial homeostasis, and inflammation-dependent oxidative stress [15, 29, 31, 33, 34, 52, 77]. Further studies using endothelial-specific models are needed to determine whether targeting neddylation can restore redox balance and mitochondrial function in diabetic vascular disease.

## KNOWLEDGE GAPS AND ENDOTHELIAL HETEROGENEITY

Although accumulating evidence supports a role for neddylation in endothelial biology and diabetes-associated vascular injury, several important knowledge gaps remain. Most mechanistic studies directly linking dysregulated neddylation to endothelial dysfunction have been performed in retinal ECs and diabetic retinopathy models, where increased cullin neddylation promotes degradation of protective factors such as NRF2 and hnRNPA2B1, resulting in enhanced oxidative stress, inflammation, vascular leakage, and pathological angiogenesis [18, 47, 48]. While these studies provide compelling proof-of-concept evidence that aberrant neddylation can contribute to endothelial injury under diabetic conditions, it remains unclear whether similar mechanisms operate in other vascular beds. Furthermore, a substantial proportion of the evidence linking neddylation to diabetes-related metabolic dysfunction has been derived from studies in hepatocytes, adipocytes, macrophages, skeletal muscle, and cancer cells rather than ECs [29–35, 49–52]. Consequently, direct evidence establishing endothelial-specific neddylation signaling as a causal determinant of diabetic vascular dysfunction remains limited.

An additional challenge arises from the remarkable heterogeneity of ECs across the vascular system. ECs exhibit substantial organ-specific differences in morphology, gene expression, metabolic activity, permeability, angiogenic capacity, and responses to pathological stimuli. Recent advances in single-cell transcriptomic analyses have revealed distinct endothelial phenotypes in the retina, heart, kidney, brain, lung, liver, and peripheral vasculature, indicating that endothelial responses to metabolic stress are highly context dependent [7, 68]. The retinal endothelium is characterized by specialized barrier properties that contribute to the blood-retinal barrier, whereas glomerular ECs are highly fenestrated and coronary ECs are uniquely adapted to dynamic metabolic and mechanical demands [7, 68]. Therefore, molecular mechanisms identified in retinal ECs may not fully reflect the biology of endothelial dysfunction in coronary artery disease, peripheral artery disease, diabetic nephropathy, or diabetic cardiomyopathy.

The potential impact of endothelial heterogeneity is particularly relevant to neddylation signaling because many neddylation-regulated pathways exhibit strong tissue-specific and context-dependent effects. For example, CUL3-dependent regulation of NRF2 signaling plays a central role in diabetic retinopathy, where increased neddylation promotes oxidative stress through enhanced NRF2 degradation [18, 48]. In contrast, hepatocyte-specific deletion of CUL3 results in excessive NRF2 activation, NADH reductive stress, mitochondrial dysfunction, and systemic insulin resistance, highlighting the complexity of neddylation-dependent redox regulation across tissues [29]. Similarly, physiological neddylation is required for endothelial barrier integrity, angiogenesis, vascular remodeling, and VEGFR2 stability, whereas excessive neddylation promotes endothelial inflammation and vascular injury [40, 41, 44–46, 74]. These observations suggest that both insufficient and excessive neddylation may have detrimental consequences depending on cell type, disease stage, and microenvironmental conditions.

Another important unanswered question is whether dysregulated neddylation contributes directly to macrovascular complications of diabetes. Endothelial dysfunction is a central pathogenic mechanism underlying atherosclerosis, coronary artery disease, peripheral artery disease, cerebral vascular disease, and heart failure, yet direct investigations of neddylation in these conditions remain scarce [13, 14, 68]. Existing evidence from oxidized low-density lipoprotein-induced endothelial injury and inflammatory vascular models suggests that excessive neddylation can impair endothelial NO signaling and enhance inflammatory activation [56, 74, 75]. However, endothelial-specific genetic studies examining the role of NEDD8, NAE1, UBE2M, UBE2F, or cullin family members in diabetic macrovascular disease are largely lacking. Consequently, whether the mechanisms identified in retinal ECs can be generalized to systemic vascular complications remains unknown.

In addition, several emerging mechanisms that contribute to diabetic endothelial dysfunction have not been explored in the context of neddylation. Recent studies highlight important roles for endothelial senescence, extracellular vesicle-mediated intercellular communication, epigenetic regulation, endothelial-to-mesenchymal transition, mitochondrial quality control, and impaired vascular repair in diabetic vascular disease [46, 70–72]. Although neddylation has been implicated in cellular senescence, mitochondrial dynamics, autophagy, and chromatin regulation [15, 46, 49, 94, 95], whether these pathways converge to regulate endothelial dysfunction under diabetic conditions remains largely unexplored. Elucidating these interactions may provide important insights into how neddylation integrates metabolic stress signals to influence vascular homeostasis.

Future studies employing endothelial-specific gain- and loss-of-function models, single-cell transcriptomics, spatial transcriptomics, multiomic approaches, and vascular bed-specific analyses will be essential for defining the precise role of neddylation in diabetic vascular disease. Such investigations should determine whether dysregulated neddylation represents a common mechanism underlying endothelial dysfunction across multiple organs or whether its effects are restricted to specific endothelial subpopulations. Addressing these knowledge gaps will be critical for evaluating the therapeutic potential of targeting neddylation and for developing tissue-specific strategies aimed at preventing diabetes-associated vascular complications [7, 68, 99].

## THERAPEUTIC TARGETING OF THE NEDDYLATION PATHWAY IN DIABETES-ASSOCIATED ENDOTHELIAL DYSFUNCTION

The growing recognition of neddylation as a regulator of metabolism, inflammation, oxidative stress, mitochondrial homeostasis, and endothelial function has generated interest in targeting the NEDD8 pathway as a therapeutic strategy for diabetes-associated vascular disease [15, 28, 34, 100]. Because neddylation regulates multiple upstream drivers of endothelial dysfunction, including hyperglycemia, insulin resistance, inflammation, oxidative stress, and mitochondrial dysfunction, modulation of this pathway may provide broad vascular protection [15, 28, 34, 35, 37, 101].

### Global inhibition of the neddylation pathway

The most extensively studied approach is inhibition of the NEDD8-activating enzyme (NAE). MLN4924 (pevonedistat), the first-in-class NAE inhibitor, blocks NEDD8 activation and suppresses global neddylation signaling [38, 39]. Although originally developed for oncology, MLN4924 has demonstrated beneficial metabolic effects in preclinical models, including enhanced hepatic insulin signaling through stabilization of insulin receptor substrate proteins, reduced hyperglycemia, suppression of inflammatory cytokine production, activation of NRF2-dependent antioxidant defenses, and improved mitochondrial fatty acid oxidation [35, 52, 88, 96–98]. Collectively, these findings suggest that NAE inhibition may attenuate several pathogenic processes implicated in diabetic endothelial dysfunction.

Moreover, because MLN4924 was originally developed as an anticancer agent, many of its biological effects are mediated through antiproliferative and cytotoxic mechanisms. Although such properties may be beneficial in oncology, they could potentially impair endothelial survival, physiological angiogenesis, and vascular repair, processes that are essential for maintaining vascular homeostasis and recovery from diabetic vascular injury. In addition, despite encouraging preclinical findings, clinical translation of NEDD8 pathway inhibition has proven challenging. The phase III PANTHER trial evaluating pevonedistat plus azacitidine in higher-risk myeloid malignancies failed to demonstrate a significant improvement in its primary endpoint of event-free survival, underscoring the complexity of translating neddylation-targeted therapies into clinical benefit and highlighting the need for continued optimization of therapeutic strategies [102].

However, global inhibition of neddylation also presents important challenges. Clinical studies have reported dose-limiting toxicities, including fatigue, gastrointestinal symptoms, cytopenias, hepatotoxicity, and increased susceptibility to infection [103, 104]. Moreover, physiological neddylation is required for endothelial barrier integrity, angiogenesis, mitochondrial function, vascular repair, and VEGFR2 stability [40, 44, 45]. Consequently, prolonged suppression of neddylation may impair normal endothelial function, emphasizing the need for careful dose optimization and context-specific therapeutic strategies [104]. A second-generation NAE inhibitor, TAS4464, exhibits greater potency and selectivity than MLN4924 and may provide an alternative platform for future investigation in metabolic disease [57, 59].

### Selective and cell-specific approaches

Given the essential physiological functions of neddylation, increasing efforts are focused on the selective targeting of individual pathway components. UBE2M is particularly attractive because it regulates cullin neddylation and has been implicated in obesity-associated inflammation, insulin resistance, endothelial senescence, and VEGFR2 stabilization [31, 46]. Macrophage-specific deletion of UBE2M improves obesity-associated metabolic dysfunction in experimental models, supporting the concept that selective pathway modulation may provide metabolic benefits while avoiding complete suppression of neddylation [31]. Additional targets include UBE2F and DCN1-dependent pathways, which may offer greater specificity and reduced toxicity compared with global NAE inhibition [23, 25, 26, 31, 58, 105].

Emerging precision-therapy approaches may further improve therapeutic selectivity. Nanoparticle-based delivery systems have demonstrated the feasibility of suppressing neddylation within specific cell populations while limiting systemic exposure [37]. Similarly, extracellular vesicle-based approaches have shown efficacy in diabetic retinopathy through suppression of pathological CUL3 neddylation and restoration of NRF2 signaling [59]. These findings raise the possibility that endothelial-targeted or cell-specific modulation of neddylation could suppress pathological signaling while preserving physiological pathways required for vascular repair and homeostasis [18, 46–48, 68].

### Future perspectives

Current evidence supports dysregulated neddylation as a potential therapeutic target in diabetes-associated endothelial dysfunction. Nevertheless, the dual role of neddylation in both physiological and pathological processes suggests that future strategies should prioritize tissue-specific, cell-specific, or substrate-selective approaches rather than complete pathway inhibition. Such precision therapies may ultimately provide safer and more effective interventions for preventing diabetic vascular complications [15, 27, 34, 100, 106].

## CONCLUSIONS AND FUTURE PERSPECTIVES

Endothelial dysfunction is a fundamental pathological feature of T2D and a critical link between metabolic abnormalities and the development of diabetic vascular complications. Chronic hyperglycemia, insulin resistance, obesity-associated inflammation, oxidative stress, and mitochondrial dysfunction converge to disrupt endothelial homeostasis, leading to impaired NO bioavailability, inflammatory activation, barrier dysfunction, endothelial senescence, and defective vascular repair [13, 14, 62, 63, 68]. Although substantial progress has been made in understanding these processes, the molecular mechanisms that integrate metabolic stress with endothelial injury remain incompletely defined.

The evidence reviewed herein supports the concept that dysregulated neddylation functions as a molecular integrator linking metabolic stress to endothelial dysfunction in T2D. Through modification of both cullin and non-cullin substrates, neddylation regulates diverse pathways involved in protein turnover, insulin signaling, inflammation, redox homeostasis, mitochondrial function, angiogenesis, endothelial senescence, and cellular adaptation to stress [15, 17, 27, 28]. Emerging studies suggest that aberrant neddylation contributes to major metabolic drivers of endothelial dysfunction, including hyperglycemia, insulin resistance, obesity-associated metabolic dysfunction, oxidative stress, and mitochondrial abnormalities [29–35, 49–52]. Importantly, studies in diabetic retinopathy demonstrate that excessive neddylation promotes endothelial injury through degradation of protective factors such as NRF2 and hnRNPA2B1, whereas physiological neddylation is required for maintenance of endothelial homeostasis, in part through stabilization of VEGFR2 and preservation of endothelial regenerative capacity [18, 46–48]. Together, these findings indicate that the vascular consequences of neddylation are highly context-dependent and that both excessive and insufficient neddylation may adversely affect endothelial health.

These observations place neddylation at the intersection of multiple pathways that govern endothelial function and vascular homeostasis in diabetes. Many neddylation-regulated pathways—including NRF2-dependent antioxidant signaling, inflammatory responses, insulin receptor substrate stability, mitochondrial quality control, VEGFR2 signaling, and angiogenic regulation—are key determinants of endothelial health and vascular integrity [15, 18, 29–39, 49–52]. This convergence supports a role for dysregulated neddylation as a common upstream contributor to diabetic vascular complications.

Pharmacological targeting of the neddylation pathway has therefore emerged as a promising therapeutic strategy. Inhibition of NEDD8 activation with agents such as MLN4924 and TAS4464 improves insulin sensitivity, reduces hyperglycemia, suppresses inflammation, enhances antioxidant defenses, and attenuates tissue injury in experimental models [33, 35, 37, 52, 88, 96, 97, 101]. Beyond global NAE inhibition, emerging approaches include selective targeting of UBE2M-, UBE2F-, and DCN1-dependent pathways, as well as nanoparticle-based, endothelial-targeted, and extracellular vesicle-mediated delivery systems [6, 25, 26, 31]. These strategies may ultimately enable suppression of pathological neddylation while preserving physiological functions required for endothelial maintenance, angiogenesis, and vascular repair.

Despite these advances, several important questions remain unresolved. Most mechanistic evidence directly linking neddylation to endothelial dysfunction derives from retinal ECs and diabetic retinopathy models, limiting the generalizability of current findings to other vascular beds and diabetic complications [18, 47, 48, 68]. Whether similar mechanisms operate in coronary artery disease, peripheral artery disease, diabetic nephropathy, and cerebrovascular disease remains unknown [68]. In addition, much of the evidence implicating neddylation in metabolic dysfunction comes from studies in hepatocytes, adipocytes, macrophages, skeletal muscle cells, and cancer cells rather than ECs themselves [29–35, 46, 49–52, 72, 107]. Future studies should define endothelial-specific neddylation substrates, determine how endothelial heterogeneity influences neddylation signaling across vascular beds, and clarify the roles of neddylation in mitochondrial quality control, extracellular vesicle-mediated communication, epigenetic remodeling, senescence, and vascular repair [46, 72, 80, 91, 104].

In summary, neddylation has emerged as a previously underappreciated regulator of endothelial biology and cardiometabolic homeostasis. Although direct endothelial evidence remains limited and currently derives predominantly from retinal endothelial cells and diabetic retinopathy models, accumulating data support the hypothesis that dysregulated neddylation may function as a molecular link between metabolic dysfunction and vascular injury in T2D. A more comprehensive understanding of endothelial neddylation signaling will not only advance our knowledge of diabetic vascular pathogenesis but may also facilitate the development of precision therapeutic strategies aimed at preventing or reversing diabetes-associated cardiovascular and microvascular complications (Fig. 3).

## Figures and Tables

**Fig. 1:**
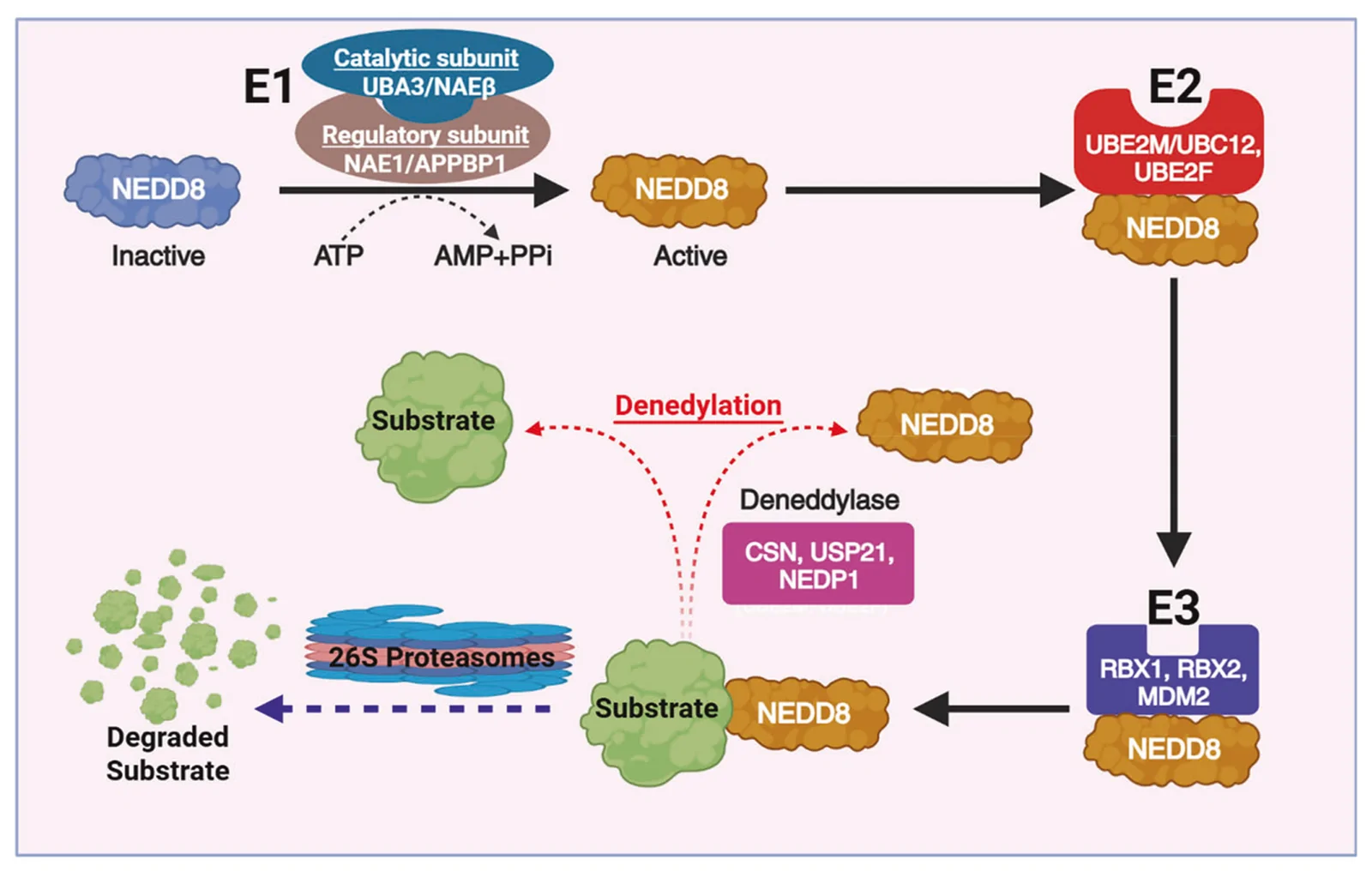
Schematic overview of the neddylation and deneddylation pathways. Neddylation is a reversible post-translational modification in which NEDD8 is covalently conjugated to substrate proteins through a sequential enzymatic cascade. In the activation step, inactive NEDD8 is activated in an ATP-dependent manner by the heterodimeric E1 NEDD8-activating enzyme (NAE), consisting of the catalytic subunit UBA3 (NAEß) and the regulatory subunit NAE1 (APPBP1), generating a high-energy NEDD8-E1 intermediate. Activated NEDD8 is subsequently transferred to the E2 conjugating enzymes ubiquitin-conjugating enzyme E2 M (UBE2M/UBC12) or ubiquitin-conjugating enzyme E2 F (UBE2F) and then ligated to specific substrate proteins by E3 ligases, including ring-box 1 (RBX1), ring-box 2 (RBX2/RNF7), and murine double minute 2 (MDM2). Conjugation of NEDD8 alters substrate stability, activity, localization, and protein-protein interactions, thereby regulating diverse cellular processes. Neddylation is dynamically reversed by deneddylases, including the COP9 signalosome (CSN), ubiquitin-specific protease 21 (USP21), and NEDD8-specific protease 1 (NEDP1/SENP8), which remove NEDD8 from substrates and recycle free NEDD8. Depending on the substrate and biological context, neddylation may influence protein degradation and turnover through proteasome-dependent mechanisms, thereby contributing to the maintenance of cellular homeostasis. APPBP1 amyloid precursor protein-binding protein 1, CSN COP9 signalosome, MDM2 murine double minute 2, NEDD8 neural precursor cell expressed, developmentally downregulated 8, NAE NEDD8-activating enzyme, NAE1 NEDD8-activating enzyme subunit 1, NEDP1 NEDD8-specific protease 1, UBA3 ubiquitin-like modifier activating enzyme 3, UBC12 ubiquitin-conjugating enzyme 12, UBE2F ubiquitin-conjugating enzyme E2 F, UBE2M ubiquitin-conjugating enzyme E2 M, RBX1, ring box 1 RBX2, ring-box 2, RNF7 ring finger protein 7, USP21 ubiquitin-specific protease 21 (Figure is generated using Biorender).

**Fig. 2:**
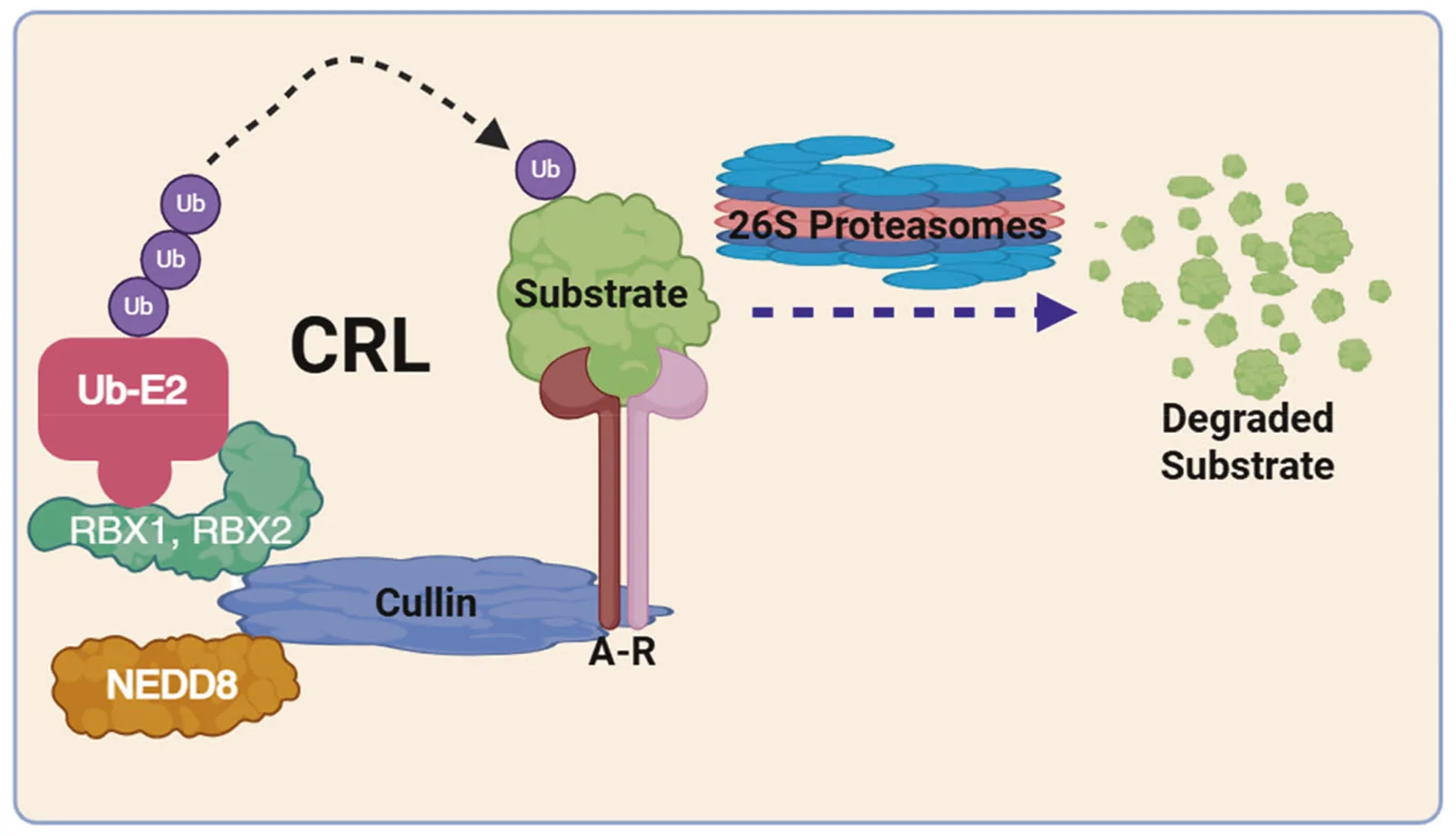
Schematic illustration of CRL-mediated ubiquitination and proteasomal degradation. Cullin-RING ligases (CRLs) are multi-subunit E3 ubiquitin ligase complexes composed of a cullin scaffold, RBX1/2 (RING-box proteins), and adapter-receptor modules that confer substrate specificity. NEDD8 conjugation to cullin (neddylation) activates the CRL complex, promoting recruitment of ubiquitin-loaded E2 enzymes (Ub-E2). The substrate is recognized by A-R (adapter-receptor complex) and undergoes polyubiquitination. Polyubiquitinated substrates are subsequently targeted to the 26S proteasome for degradation, resulting in substrate breakdown into smaller peptide fragments. A-R adapter/substrate receptor protein complex, CRL Cullin-RING ligase, Ub ubiquitin, NEDD8 neural precursor cell expressed, developmentally downregulated 8, U RBX1 ring-box 1, RBX2 ring-box 2 (RNF7/SAG), b-E2 ubiquitin-charged E2 conjugating enzyme, 26S proteasome 26S proteasomal degradation complex (Figure is generated using Biorender).

**Fig. 3:**
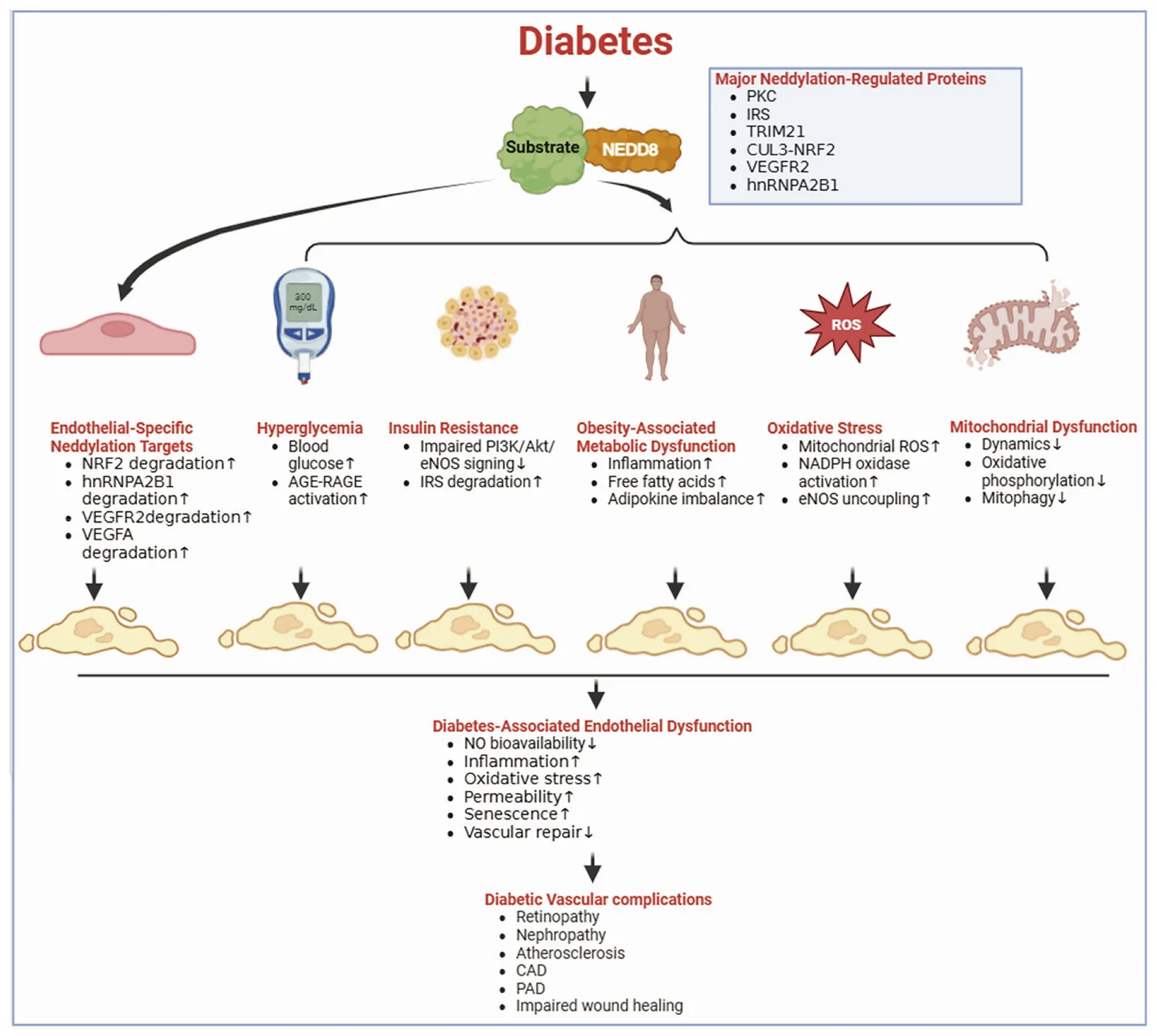
Schematic illustration of the proposed mechanisms by which dysregulated neddylation contributes to diabetes-associated endothelial dysfunction. Diabetes and diabetes-associated metabolic stress may enhance neddylation signaling, leading to alterations in multiple interconnected pathways implicated in vascular injury. Emerging evidence suggests that dysregulated neddylation promotes hyperglycemia through regulation of glucose metabolic enzymes, including PCK1, impairs insulin signaling through modulation of IRS stability, and contributes to obesity-associated inflammation through the UBE2M-TRIM21-HIF-1α signaling axis. Neddylation also regulates oxidative stress through the CUL3-KEAP1-NRF2 pathway and influences mitochondrial homeostasis by modulating mitochondrial dynamics, mitophagy, and bioenergetic function. In endothelial cells, excessive neddylation promotes degradation of protective factors, including NRF2 and hnRNPA2B1, resulting in increased oxidative stress, inflammation, vascular leakage, and impaired endothelial function. In contrast, physiological neddylation contributes to endothelial homeostasis through stabilization of VEGFR2, thereby supporting endothelial survival, angiogenesis, and resistance to senescence. Collectively, these mechanisms converge to reduce nitric oxide NO bioavailability, enhance oxidative stress and inflammatory signaling, impair vascular repair, and promote endothelial dysfunction, ultimately contributing to the development of diabetic microvascular and macrovascular complications. AGEs advanced glycation end products, eNOS endothelial nitric oxide synthase, HIF-1α hypoxia-inducible factor-1α, hnRNPA2B1 heterogeneous nuclear ribonucleoprotein A2/B1, IRS insulin receptor substrate, KEAP1 Kelch-like ECH-associated protein 1, NEDD8 neural precursor cell expressed, developmentally downregulated 8, NO nitric oxide, NRF2 nuclear factor erythroid 2-related factor 2, PCK1 phosphoenolpyruvate carboxykinase 1, PI3K phosphoinositide 3-kinase, ROS reactive oxygen species, TRIM21 tripartite motif-containing protein 21, UBE2M ubiquitin-conjugating enzyme E2M, VEGFR2 vascular endothelial growth factor receptor 2 (Figure is generated using Biorender).

**Table 1.** Studies linking dysregulated neddylation to endothelial dysfunction in non-diabetic and diabetic vascular disease and their potential therapeutic implications.

Disease/model	Neddylation target/pathway	Mechanism	Endothelial outcome	Therapeutic implication	Study
OxLDL-induced vascular injury (non-diabetic)	Increased protein neddylation; HDAC2 degradation	Enhanced neddylation promotes HDAC2 degradation, resulting in increased arginase-2 expression and reduced eNOS-derived NO production	Impaired endothelium-dependent vasorelaxation and endothelial dysfunction; MLN4924 restored endothelial function	Neddylation inhibition (e.g., MLN4924) may restore NO bioavailability and improve endothelial function	[74]
Endothelial inflammatory response	SENP8 (NEDP1)-regulated deneddylation	Loss of deneddylation alters Cullin-dependent inflammatory signaling	Enhanced endothelial inflammatory responses	Restoration of deneddylation pathways may suppress endothelial inflammation	[56]
Vascular inflammation	CSN5-mediated deneddylation	CSN5 suppresses NF-κB activation and monocyte adhesion	Reduced endothelial inflammation and leukocyte recruitment	Enhancing CSN5 activity may attenuate vascular inflammatory responses	[75]
Mucosal inflammatory injury	Cullin-2 neddylation/HIF pathway	Excessive cullin activity promotes inflammatory signaling	Neddylation inhibition stabilizes HIF and attenuates vascular inflammation	Targeting Cullin-2 neddylation may provide anti-inflammatory vascular protection	[42]
Diabetic retinopathy	CUL3-mediated NRF2 degradation	Increased CUL3 neddylation promotes NRF2 degradation, suppressing antioxidant defenses	Oxidative stress, inflammation, blood-retinal barrier disruption, and retinal endothelial injury	Inhibition of CUL3 neddylation or stabilization of NRF2 may protect against diabetic retinal vascular injury	[18]
Diabetic retinopathy	CUL3 neddylation–NRF2 pathway	miR-143-3p inhibits CUL3 neddylation and stabilizes NRF2	Reduced ROS accumulation and protection against retinal endothelial dysfunction	Extracellular vesicle- or miRNA-based targeting of CUL3 neddylation may represent a novel therapeutic strategy	[48]
Diabetic retinopathy; high glucose-treated retinal ECs	hnRNPA2B1 degradation via neddylation	Neddylation-mediated degradation of hnRNPA2B1 disrupts the miR-93-5p/VEGFA axis	Endothelial dysfunction, vascular leakage, and pathological angiogenesis	Preservation of hnRNPA2B1 signaling may prevent retinal endothelial dysfunction and vascular leakage	[47]
Pulmonary vascular ECs	UBE2M-mediated VEGFR2 neddylation	Neddylation stabilizes VEGFR2 and preserves VEGFR2 signaling	Delayed endothelial senescence; maintenance of endothelial proliferation, angiogenic capacity, and vascular homeostasis	Therapeutic strategies should preserve physiological VEGFR2 neddylation while selectively inhibiting pathological neddylation events	[46]
